# Comparing Quantitative Values of Two Generations of Laser-Assisted Indocyanine Green Dye Angiography Systems: Can We Predict Necrosis?

**Published:** 2014-12-05

**Authors:** Brett T. Phillips, Mitchell S. Fourman, Andrew Rivara, Alexander B. Dagum, Tara L. Huston, Jason C. Ganz, Duc T. Bui, Sami U. Khan

**Affiliations:** ^a^Division of Plastic, Maxillofacial, & Oral Surgery, Duke University Medical Center, Durham, NC; ^b^Department of Orthopaedic Surgery, University of Pittsburgh Medical Center, Pittsburgh, PA; ^c^Stony Brook University School of Medicine, Stony Brook, NY; ^d^Stony Brook University Medical Center, Division of Plastic and Reconstructive Surgery

**Keywords:** breast reconstruction, indocyanine green dye, mastectomy skin flap necrosis, quantitative perfusion values, SPY

## Abstract

**Objective:** Several devices exist today to assist the intraoperative determination of skin flap perfusion. Laser-Assisted Indocyanine Green Dye Angiography (LAICGA) has been shown to accurately predict mastectomy skin flap necrosis using quantitative perfusion values. The laser properties of the latest LAICGA device (SPY Elite) differ significantly from its predecessor system (SPY 2001), preventing direct translation of previous published data. The purpose of this study was to establish a mathematical relationship of perfusion values between these 2 devices. **Methods:** Breast reconstruction patients were prospectively enrolled into a clinical trial where skin flap evaluation and excision was based on quantitative SPY Q values previously established in the literature. Initial study patients underwent mastectomy skin flap evaluation using both SPY systems simultaneously. Absolute perfusion unit (APU) values at identical locations on the breast were then compared graphically. **Results:** 210 data points were identified on the same patients (n = 4) using both SPY systems. A linear relationship (*y* = 2.9883*x* + 12.726) was identified with a high level or correlation (R^2^ = 0.744). Previously published values using SPY 2001 (APU 3.7) provided a value of 23.8 APU on the SPY Elite. In addition, postoperative necrosis in these patients correlated to regions of skin identified with the SPY Elite with APU less than 23.8. **Conclusion:** Intraoperative comparison of LAICGA systems has provided direct correlation of perfusion values predictive of necrosis that were previously established in the literature. An APU value of 3.7 from the SPY 2001 correlates to a SPY Elite APU value of 23.8.

More than 93,000 breast reconstructions were performed in 2011, with overall complication rates reported to be as high as 60%.^1^^,^^2^ All inclusive mastectomy skin flap (MSF) necrosis can complicate more than 40% of implant-based breast reconstructions following simple mastectomy.^3^^,^^4^ Mastectomy skin flap necrosis can result in infection, delayed oncologic therapy, and reconstructive failure. The intraoperative gold standard approach to diagnosis of viable MSFs includes surgeon assessment of skin flap color, capillary refill, temperature, turgor, and skin edge bleeding. Two widely studied methods that can aid in the determination of flap viability include fluorescein dye angiography (FDA) and laser-assisted indocyanine green dye angiography (LAICGA).^5^^-^7 Both methods involve intraoperative injection of dye followed by illumination with a Wood's Lamp or Near Infrared Light, respectively. These adjunctive techniques have been shown to determine areas of ischemia that may ultimately result in skin necrosis.^3^^-^8

A recent study performed by our institution compared these 3 methods of clinical assessment, FDA and LAICGA in predicting MSF necrosis in breast reconstruction.^3^ This prospective study concluded that LAICGA was a better predictor of mastectomy skin flap necrosis when compared to either FDA or clinical assessment. Laser-assisted indocyanine green dye angiography was even more predictive and accurate when employing the quantitative values provided by the LAICGA SPY Q software. The predictive values determined by this previous study were determined using an earlier model LAICGA system (SPY 2001) that does not directly translate to the latest device (SPY Elite). The options for effectively translating quantitative values from one system to another included repeating the previous study or completing a direct comparison of both machines. In order to avoid repeating our work, the goal of this study was to simultaneously compare quantitative values determined by both SPY 2001 and SPY Elite on the same breast reconstruction patient.

## PATIENTS AND METHODS

A prospective case series was designed to compare 2 different LAICGA systems to predict MSF necrosis in immediate breast reconstruction (IBR). This patient series received institutional review board's approval prior to enrollment. Inclusion criteria included all patients undergoing prophylactic or therapeutic mastectomy followed by IBR at our institution. Exclusion criteria included delayed reconstruction, iodine allergy, and liver failure. Our primary outcome was all-inclusive MSF necrosis and the corresponding SPY Q values. Baseline patient demographics were also recorded.

### Operation

Mastectomy was performed by 1 of 4 breast surgeons followed by reconstruction by 1 of 2 plastic surgeons. No tumescence, local anesthetic, or epinephrine-containing injections were used prior to or during surgery. Immediate breast reconstruction was conducted using a tissue expander with an acellular dermal matrix or free transverse rectus abdominis myocutaneous flap. Imaging was performed at a minimum of 1-hour postmastectomy and prior to completion of reconstruction. Mastectomy skin flaps were assessed and excised based on clinical judgment. The area of excision was not the primary focus of this study and was therefore not measured. Postoperative photo documentation was performed to confirm predictive value of SPY Q numbers.

### SPY protocol

**Figure 1:**
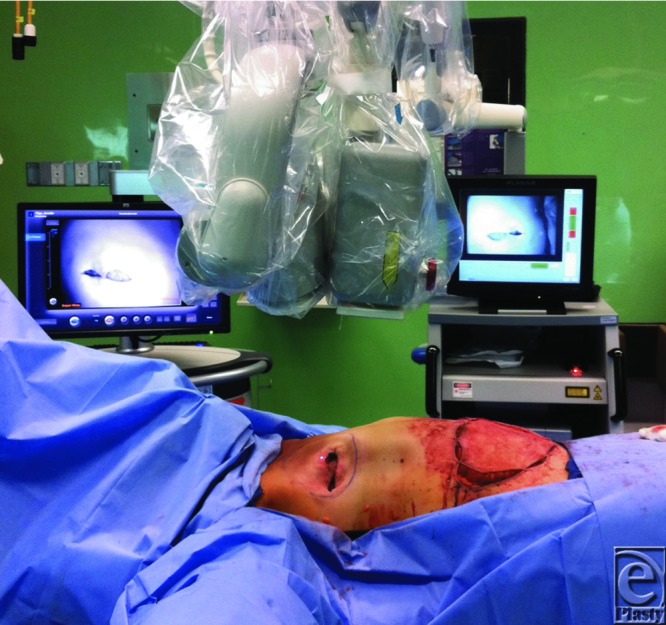
Intraoperative setup of dual machines for comparison. SPY Elite (*left*), SPY 2001 (*right*).

Each patient received a 10-mg intravenous dose of indocyanine green with illumination of MSFs using the SPY Elite Imaging System (Lifecell Corp, Branchburg, New Jersey) and the SPY 2001 Imaging System (Novadaq, Bonita Springs, Florida). Both machines were set up over the mastectomy skin flap region of interest prior to injection as seen in Figure 1 and were not moved. Image recording began with the first blush of dye using the SPY Elite System. At 2 minutes postinjection, the SPY Elite System was stopped and the SPY 2001 device was started simultaneously. The 2-minute analysis time point was used to be consistent with our previous protocol.^3^

### SPY devices

**Figure 2:**
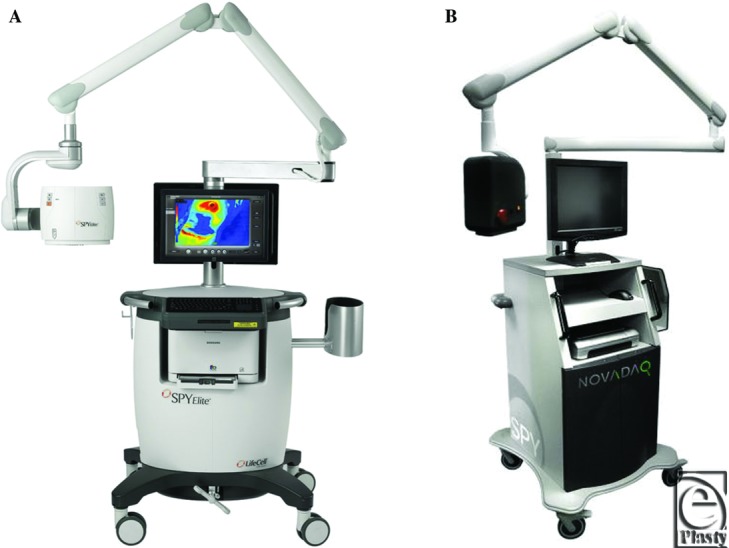
(*a*) SPY Elite Imaging System (Lifecell), (*b*) SPY 2001 Imaging System (Novadaq).

The SPY 2001 device is a previous generation model near infrared laser and fluorescence capturing system. The decreased power of its laser and ambient light filtration requires increased dosages of indocyanine green and necessitates the operating room lights to be turned off. In addition, its recording system only allows for imaging at 1-minute increments. In contrast, the SPY Elite Imaging system, though sharing similar overall properties, is the latest device with a more powerful laser and an improved operating system. Using this updated system, overhead lights can be kept on, less dye is required, and longer recording times of up to 4 minutes are possible. The general layout of each device is similar with a central computer base, monitor, and adjustable laser head unit (Figs. 2A, B).

### Analysis

The recordings from each SPY device were evaluated using the same SPY Q software available on each system. A single frame from each recording can then be assessed using this program and absolute perfusion units (APU) applied to target areas. The SPY Q program can interpret perfusion values using relative perfusion units (RPU) and APU. Relative perfusion units provide a percentage of perfusion at the region of interest based on a target area identified by the software or surgeon as being the “100% perfusion marker.” Absolute perfusion unit is based on a standardized scale of gray pixel shades from 0 to 255. Values closer to zero exhibit little to no perfusion while higher numbers signal increased perfusion. Inanimate objects within the scanning field will exhibit perfusion values of zero. We chose to use APU based on our previous experience.

The last frame from the SPY Elite was directly compared to the first frame of the SPY 2001 System. Equivalent locations were subjectively identified throughout the region of interest by 2 different authors. The corresponding values were then graphically represented using Excel (Microsoft Corp, Redmond, Washington) in order to provide a mathematical correlation. A best-fit line was then determined and given a R-squared value. The previously published 90% sensitive, 100% specific, MSF necrosis value (APU = 3.7) from the SPY 2001 was then applied to ascertain the same predictive value using the SPY Elite [3].

## RESULTS

Four patients were included in this comparative study with an average age of 46 (range: 37-54) and mean BMI of 31.1 (range: 25.9-45.9). All patients underwent IBR with a free transverse rectus abdominis myocutaneous flap (n = 1) or tissue expander placement with acellular dermal matrix (n = 3).

**Figure 3:**
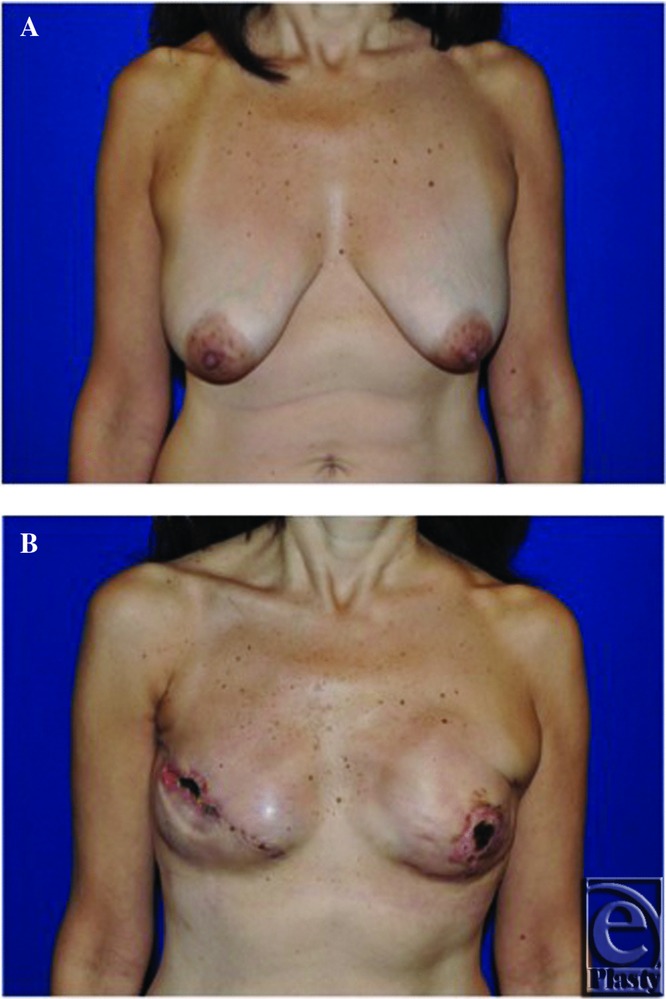
(*a*) Patient 1 preoperative picture, (*b*) Postoperative picture after left-sided nipple sparing immediate breast reconstruction with mastectomy skin flap and nipple necrosis.

**Figure 4:**
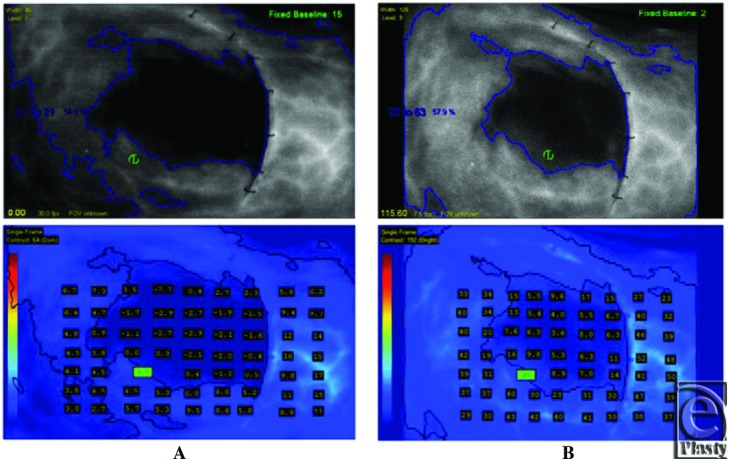
(*a*) Patient 1 intraoperative SPY Q analysis using SPY 2001; (*b*) Intraoperative SPY Q analysis using SPY Elite.

**Figure 5:**
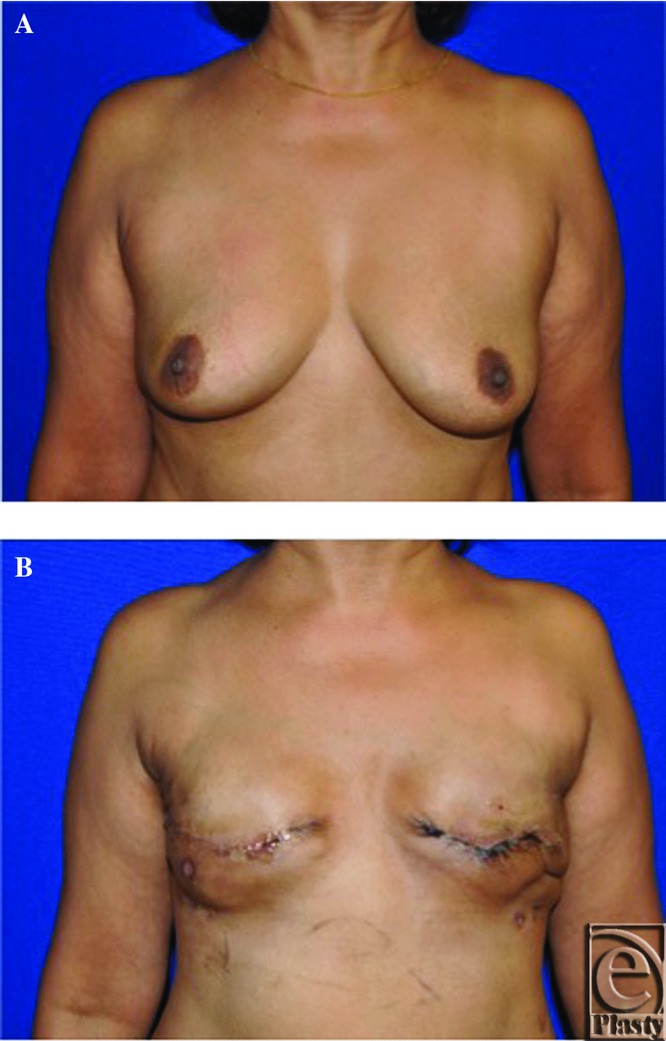
(*a*) Patient 2 preoperative picture, (*b*) Postoperative picture after immediate breast reconstruction with incisional necrosis.

**Figure 6:**
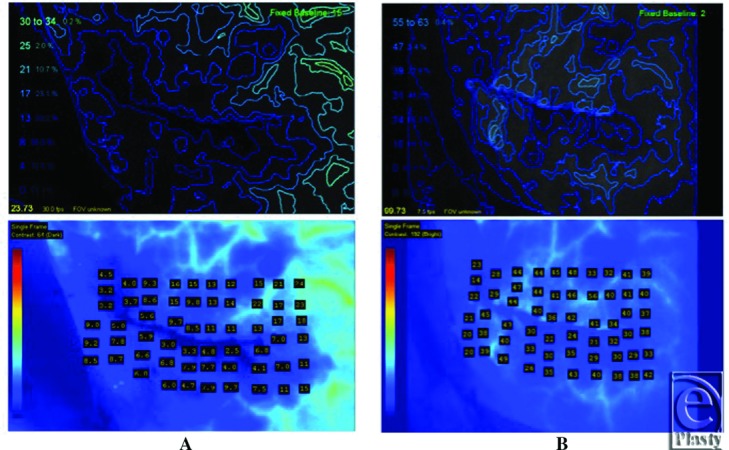
(*a*) Patient 2 intraoperative SPY Q analysis using SPY 2001; (*b*) Intraoperative SPY Q analysis using SPY Elite.

Three of the 4 patients developed some evidence of superficial or full-thickness skin flap necrosis. SPY Q values of less than 3.7 recorded by the SPY 2001 correlated with each episode of necrosis, maintaining consistency with previously published results. The first patient underwent a nipple-sparing mastectomy and subsequently developed full-thickness necrosis (Fig 3). Intraoperative perfusion values were consistent with necrosis as determined by the SPY 2001 quantitative analysis (Fig 4). The second patient with SPY Q values of less than 3.7 APU during intraoperative analysis went on to develop superficial incisional necrosis (Figs 5 and 6).

**Figure 7:**
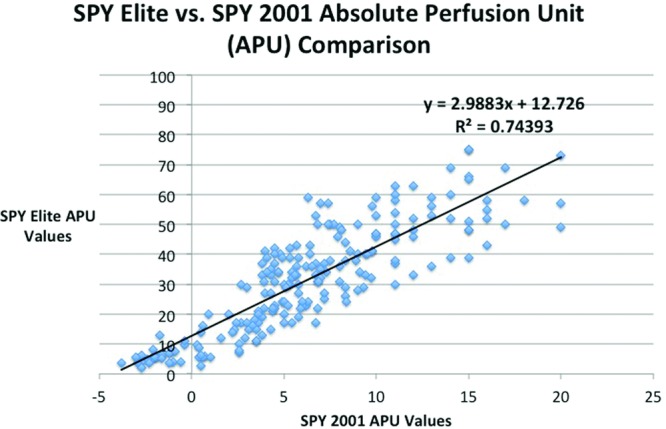
Scatter plot graph comparing SPY 2001 (*x* axis) and SPY Elite (*y* axis) absolute perfusion unit (APU) values. A best-fit line was created with equation and R-squared value.

We identified 210 APU data points on 4 patients using both SPY systems and SPY Q analyses. A linear relationship (*y* = 2.9883*x* + 12.726) was identified using a best-fit line with a high level of correlation represented by a R^2^ value of 0.744 (Fig 7). Previously published APU values using SPY 2001 (APU 3.7) provided a value of 23.8 APU on the SPY Elite. In addition, postoperative necrosis in these patients correlated to regions of skin identified with the SPY Elite with APU less than 23.8. Also, previously published APU values using SPY 2001 (APU 8.0) provided a value of 36.6 APU on the SPY Elite. This corresponded to our previous studies results of 100% sensitivity and 70% specificity.

## DISCUSSION

Mastectomy skin flap necrosis is one of several frequent complications associated with an increased morbidity in breast reconstruction. Necrosis has been reported in the literature to exceed 40% and can result in wound dehiscence, surgical site infection, loss of implant, and reconstructive failure.^3^ Several adjunctive techniques have provided intraoperative clinical guidance in the diagnosis of ischemic and potentially necrotic skin flaps including FDA^5^^,^^6^ and LAICGA.^9^ Although FDA has been used since the 1970s in plastic surgery,^5^ LAICGA has increased popularity over the last 10 years. Many case series, retrospective studies, prospective studies, and reviews have been published describing its utility in random, pedicle, and free flap reconstructions.^9^^-^25

In our recent prospective trial comparing the utility of FDA and LAICGA against clinical judgment, we identified an all-inclusive 41.2% necrosis rate.^3^ Although this rate was much higher than other published studies, the prospective nature allowed us to include all patients with superficial to full-thickness necrosis. This high overall necrosis rate led to less than 10% operative interventions with an implant loss rate of only 3.9%, which is consistent with previous literature including our own prior retrospective analysis.^13^^,^^26^^,^^27^ We hypothesize that the total frequency of necrosis following breast reconstruction is likely underreported and probably represents only the cases of necrosis that require operative interventions. We also concluded that clinical judgment was inferior to LAICGA because it failed to accurately detect areas of ischemia and future necrosis. Other published studies have also shown the benefit of LAICGA in breast reconstruction in predicting intraoperative MSF necrosis.^4^^,^^7^^,^^19^

Our previous study concluded that LAICGA was more sensitive and specific especially when using the SPY-Q software.^3^ SPY-Q analysis has been reported in several studies^4^^,^^8^^,^^11^^,^^28^^,^^29^ and has been used to guide forehead flap creation and division,^11^^,^^12^ and determine perfusion characteristics of abdominal flaps.^29^ SPY Q analysis yields quantitative values that provide the surgeon with an additional measure of perfusion. Although we have now shown that a value 23.8 APU on the SPY Elite is consistent with the 3.7 APU from the older SPY 2001, an exact cutoff value is unlikely to be completely accurate in every patient. In 2013, Wu et al^13^ completed a retrospective study using the older SPY machine and found that it often underpredicted viable tissue and agreed that one distinct value was unlikely to exist with SPY-Q using relative or absolute perfusion units. Moyer and Losken decided to further describe a potential cutoff value with their retrospective study of mastectomy skin flap necrosis. They found that a gray area existed between 25% and 45% RPU. Patients with values less than 25% had nonviable tissue 90% of the time, while patients with values greater than 45% had viable tissue 98% of the time.^8^ These percentages were consistent with the quantitative values we discovered in our study when converted from APU to RPU.

Newman et al^28^ recently conducted a retrospective study looking at SPY Q analysis showing a mean relative value of 25.2 was consistent with necrosis and 43.3 correlating with adequate healing. The RPU values were consistent with values from Moyer and Phillips articles, while the absolute units were not. This further supports the unlikely ability to come up with an exact cutoff value consistent with necrosis, although a range established through future combined data sets might prove to be useful. In addition, values may be different depending on the anatomic location. Both absolute and relative units continue to be used in studies using SPY Q analysis. Because both values have proven to be useful, the ideal measurement is still up for debate.

Although an exact quantitative value might not exist universally, LAICGA has proven to be useful in decreasing postoperative complications in breast reconstruction. A recent abstract by Garvey et al examined 152 breast reconstruction patients retrospectively, comparing patients who did and did not undergo intraoperative LAICGA. The authors found significantly lower overall complications (30.4% vs 47.8%, *P* = 0.01) and lower MSF necrosis (17.4% vs 29.6%, *P* = 0.01). They concluded that LAICGA was protective, providing a 3-fold reduction in development of MSF necrosis and overall complications.^30^ Sood and Glat^31^ also performed a similar retrospective study in 91 patients and showed an approximately 2-fold higher complication rate in patients who did not have intraoperative LAICGA, although statistical significance was not reached.^31^ They did discover with statistical significance that patients without LAICGA did have a higher mean number of return visits to the operating room.

Limitations of this study include small sample size of patients and user dependence of SPY Q analysis. Only 4 patients were analyzed because we obtained a large number of comparative data points to obtain our goal of determining the mathematical relationship between the 2 SPY machines. Our 210 data points gave us a linear relationship between the 2 systems with a good correlation coefficient. Because we obtained a good correlation coefficient with the 210 data points with a linear trend, we felt that additional patients or data points were not necessary for the purpose of this study. Additional data points might have improved the correlation coefficient but would not have likely changed the overall linear relationship and obtained equation between the 2 systems. In addition, we believe that SPY Q values are used as a guide to decision making rather than providing an exact predictive value for necrosis on which to base excision. Future studies are likely to provide a range of predictive values, and this pilot study provides a base comparison between these 2 systems. Although, values less than 23.8 APU on the SPY Elite did correlate with regions of necrosis in our small patient population. Picking the exact anatomic location to determine the quantitative value on both SPY Q systems was also subjective and was done by 2 different authors to decrease bias. We also plotted absolute perfusion units instead of relative perfusion units based on our previous results.

The result of this comparison study has provided us with the relationship of SPY Q values between 2 LAICGA machines. Our results provide the ability to interpret all of the previous publications in the literature that used the SPY 2001 system. We are currently conducting a prospective trial for MSF excision based on APU less than 24 as determined using the SPY Elite Imaging System and SPY Q analysis. As LAICGA continues to expand across other surgical fields including general, colorectal, oncologic, urologic, and wound care surgery, we believe our comparison study will assist with interpreting previous SPY Q results from the previous generation SPY 2001 machine.
